# Sea Fog Dissipation Prediction in Incheon Port and Haeundae Beach Using Machine Learning and Deep Learning

**DOI:** 10.3390/s21155232

**Published:** 2021-08-02

**Authors:** Jin Hyun Han, Kuk Jin Kim, Hyun Seok Joo, Young Hyun Han, Young Taeg Kim, Seok Jae Kwon

**Affiliations:** 1 Underwater Survey Technology 21, Incheon 21999, Korea; kjkim@ust21.co.kr (K.J.K.); joohj3655@ust21.co.kr (H.S.J.); 2 Department of Bio and Brain Engineering, Korea Advanced Institute of Science and Technology, Daejeon 34141, Korea; yhhan87@kaist.ac.kr; 3 Korea Hydrographic and Oceanographic Agency, Busan 49111, Korea; sj79kwon@korea.kr

**Keywords:** sea fog dissipation, prediction of fog dissipation, machine learning, deep learning

## Abstract

Sea fog is a natural phenomenon that reduces the visibility of manned vehicles and vessels that rely on the visual interpretation of traffic. Fog clearance, also known as fog dissipation, is a relatively under-researched area when compared with fog prediction. In this work, we first analyzed meteorological observations that relate to fog dissipation in Incheon port (one of the most important ports for the South Korean economy) and Haeundae beach (the most populated and famous resort beach near Busan port). Next, we modeled fog dissipation using two separate algorithms, classification and regression, and a model with nine machine learning and three deep learning techniques. In general, the applied methods demonstrated high prediction accuracy, with extra trees and recurrent neural nets performing best in the classification task and feed-forward neural nets in the regression task.

## 1. Introduction

Sea fog is an important meteorological phenomenon, similar to wind and precipitation. It influences the daily operations of air, sea, and land transportation and has estimated negative economic effects [1]. Fog impedes visibility and is conducive to traffic crashes. A study in the USA demonstrated that depending on the state, the weather can contribute to over 21% of all crashes, and fog together with snow and rain contributed to 31,514 traffic crashes between 2000 and 2007 [2].

Sea fog is a type of fog that generally occurs from advective fog when relatively wet and warm air moves over the sea surface with its temperature falling below the dew point [3]. Sea fog can also be born onshore and be moved to the sea surface or be a mix of on- and off-shore fog [4]. In South Korea, fog occurs mostly on the west coast. It has a seasonal pattern, with most of the fog occurring during monsoon in June and July [5]. 

Fog prediction is mostly performed using either numerical or machine learning methods [6,7]. Neural networks have been used for fog prediction for a long time with varied success. Unlike other domains, fog data are relatively scarce, while neural networks work best in a data abundance regime [8,9,10]. Furthermore, fog occurs seldom and leads to a data imbalance, with most data being in the non-fog cluster.

While fog prediction is a well-studied [11] and established area, fog dissipation is scarce in the literature. Fog dissipation refers to the clearing of fog from the air and improving visibility. Some numerical approaches have been suggested to model fog dissipation. One way in which dissipation happens is when the fog droplets become larger and drop to the ground [12]. Predicting fog dissipation is important for air flight planning [13] and cargo working times [14]. 

In this paper, our goal is to find suitable machine learning and deep learning algorithms for sea fog dissipation data. The structure of this paper is as follows: in Section 2, we analyze the weather data and present a predictive modeling method; Section 3 introduces the classification and regression models used in the sea fog prediction; in Section 4, we summarize the results of the sea fog prediction performed with each model; lastly, in Section 5, the results concluded in Section 4 are further discussed. 

## 2. Weather Data and Prediction Modelling

### 2.1. Data sources and Preprocessing

**Figure 1:**
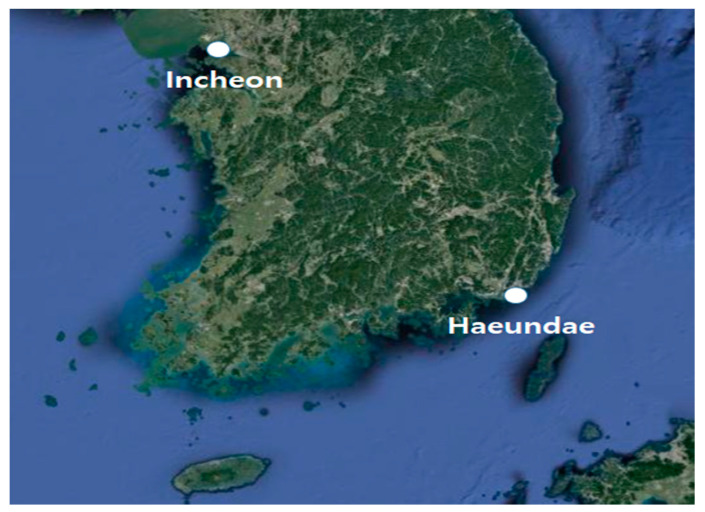
Incheon port and Haeundae beach labeled with white circles (image source: Google Maps).

Data from two sites were used: Incheon port and Haeundae beach. The observation data were obtained from the Korean Metrological Administration (KMA) and Korea Hydrographic and Oceanographic Agency (KHOA). Incheon is a strategic port in the west of the Korean Peninsula facing the Yellow Sea, while Haeundae beach is popular in the southeast of the peninsula (Figure 1).

For Incheon, the period of observations is seven years from 1 January 2012 to 31 May 2019, and for Haeundae, the period is over five years from 1 January 2014 to 31 July 2019. The objective of our study was to develop a prediction model of the sea fog dissipation to be operated on the KHOA system. The amount of sunlight in the data we collected was not considered in the preliminary feature selection, because it was not observable in real time in the KHOA system. Moreover, the cumulative precipitation was excepted, because it was confirmed to decrease the performance of the sea fog prediction when added to the learning dataset [15]. Out of the total number of variables available, seven were selected as base features: air temperature, sea surface pressure, relative humidity, sea surface temperature, visibility, u-component wind, and v-component wind. Using these given features, we created additional features: air and sea temperature difference (ASTD), dew point temperature (DT), air and dew point temperature difference (T_DT), and sea surface temperature and dew point temperature differences (sst_DT). A list of the given and created features are listed in Table 1. We resampled1-minute observations into 10-min observations. Sea surface temperature is available once in an hour, and therefore its value was assigned to six 10-minute observations.

Air and sea temperature difference, air and dew point temperature difference, sea surface temperature and dew point temperature difference are all derived by simple subtraction operation. The dew point temperature was calculated using the formula suggested in [16] as follows:

(1) $$DT=air\_temp-\left(\frac{100-humidity}{5}\right){\left(\frac{air\_temp+273.15}{300}\right)}^{2}$$

where DT is the dew temperature, air_temp is the air temperature, and *humidity* is relative humidity.

For a given time $T_{i}$ in the observed period and a feature set $f_{i}=\left[air\_temp_{i},\;sea\_air\_pre_{i},\;humidity_{i},\;sea\_temp_{i},\;vis_{i},\;u_{i},\;v_{i},\;ASTD_{i},\;DT_{i},\;T\_DT_{i},\;sst\_DT_{i}\right]$, we created the past one-hour feature vector $v1_{i}=\left[f_{i},\;f_{i-1},\;\ldots ,\;f_{i-5},\;f_{i-6}\right]$, and past three-hour feature vector $v3_{i}$ = [$f_{i},\;f_{i-1},\;\ldots ,\;f_{i-35},\;f_{i-36}$]. Then, we used these feature vectors separately to observe their effects on the training. The same set of features was used for all the models as is described in Section 2.2.

### 2.2. Dissipation as a Classification and Regression Task

Dissipation is the natural outcome of the fog. Once the fog clears and the visibility returns to normal ranges, the fog is said to have dissipated. We define fog as the visibility with less than or equal to 1100 m. The visibility may drop below or rise above this threshold at any given consecutive period. Due to this variability, fog dissipation does not always occur after the rise in visibility above the 1100 m threshold. Therefore, fog dissipation prediction is performed, even for the cases when the visibility is over 1100 m but is preceded by a fog period. 

In this work, dissipation prediction is formulated as binary classification (dissipate or non-dissipate) and regression problems. To this end, we grouped all fog cases that occur within close proximity (within one, two or three hours depending on the prediction period) into grouped fog periods of [$T_{i},\;T_{i+k}]$ consisting of k cases. The period of [$T_{i},\;T_{i+k}]$ mostly consists of cases of continual fog periods with intermittent and short non-fog cases. 

**Figure 2:**
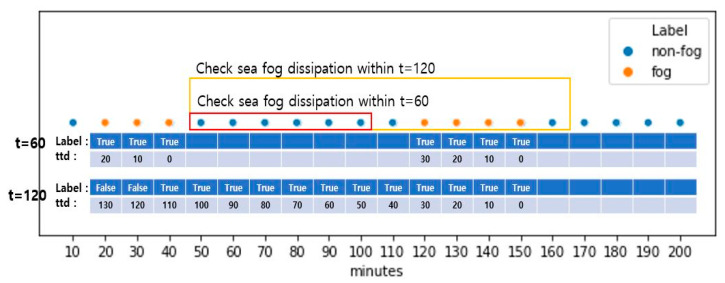
Illustration of fog and non-fog cases over the 200-min interval.

For the binary classification task, each case from the [$T_{i},\;T_{i+k}]$ period is labeled as either 1 or 0 depending on if the fog dissipates over 60, 120, or 180 min from that point. The grouping period depends on the prediction period, and for prediction horizons of 60, 120, or 180 min, the grouping period is 60, 120, or 180 min, respectively. In Figure 2, visibility dropped below 1100 m for 20, 30, 40, 120, 130, 140, and 150 min, and these are labelled as fog. When the prediction period is 60 min, and hence the same length grouping period is used, the mentioned fog occurrences are grouped into the same 20–40 and 120–150 min intervals. However, if the prediction period, and hence the grouping period of 120 min, is used, these two intervals and the interval between them are all grouped together into the 20–50 min interval. Then, the data from these intervals are included in the training. 

As can be seen, the longer the period, the more cases were grouped, and the more non-fog were cases included. In practice, this also leads to an increase in the training data for longer periods but not as linearly as the increase in the period. This is because we removed grouped cases with any missing observations in base features. In Figure 2, if any base features from the interval 20–40 are missing, then only this interval is excluded from the training, provided the interval 120–150 does not have any missing values. At the same time, when grouping and predicting for 120 min, the total 20–150 interval is excluded if any base features are missing.

The regression task aims to predict the time to dissipation for each case in the grouped [$T_{i},\;T_{i+k}]$ period. All cases in the grouped [$T_{i},\;T_{i+k}]$ period have the same dissipation time $T_{i+k+1}$, and their time to dissipation has a monotonically decreasing characteristic: {10∗k minutes, 10∗(k−1) minutes,…, 20 minutes, 10 minutes}. Thus, for a given case at index *i*∈ {i,i+1, …,i+k−1, i+k}, its time to dissipation is given:

(2) $$ttd_{i}=\left(k-i+1\right)*10minutes$$

Formally, given a prediction time period t ∈{60, 120, 180} representing 10-min intervals in 60, 120, or 180 min, a grouped fog period $\left[T_{i},\;T_{i+k}\right]$, which consists of fog cases within the prediction time proximity t from each other, is labelled $L_{i}^{t}$ as

(3) $$L_{i}^{t}=\{\begin{array}{c} True,\;\;\;\;\;\;ttd_{i}>t*10\;\;\;\;\;\;\;\;\;\;\;\;\;\;\\ False,\;\;\;\;\;\;otherwise\;\;\;\;\;\;\;\;\;\;\;\;\;\;\; \end{array}$$

 L = the dissipation within *t* hours is true; L = the maintenance within *t* hours is false.

In general, the later the case within the grouped fog period [$T_{i},\;T_{i+k}]$, the sooner the time to dissipation. Given that the longest period that we aimed to group fog cases together is three hours, we grouped fog cases that occur within three hours of proximity into distinct grouped fog periods. In practice, grouping for even longer periods yielded more non-fog cases being included in the training and, thus, presented less interest.

### 2.3. Data Characteristics 

Data came from both Incheon port and Haeundae beach (Busan) of the Republic of Korea. Incheon is located in the northwest and Haeundae beach in the southeast. These geographical differences play a role in the distribution of the base features and fog frequency. Fog cases represent 0.8% and 2.8% of all the observations for Incheon port and Haeundae beach, respectively. Therefore, the resulting number of selected data points is small despite the relatively long periods of observations. Furthermore, during the observation period from 1 January 2012 to 31 May 2019 for Incheon, and from 1 January 2014 to 31 July 2019 to Haeundae beach, 1or more of the 11features were missing, and this further led to a reduction in the size of the selected data. In such cases, the total observation period was removed, even though only one or a few observations had missing values.

Among the base features (10-minute average), air temperature, sea surface pressure, humidity, and the sea surface temperature were slightly higher in Incheon port than Haeundae beach during the observation period (Table 2). Similarly, derived features, such as air and sea temperature difference (ASTD), dew point temperature (DT), air and dew point temperature difference (T_DT), and sea surface temperature and dew point temperature difference (sst_DT), demonstrated similar characteristics. There were two optical visibility meters, with a limit of 20,000 m for the VAISALA visibility and 3000 m for the AANDERAA visibility system. The visibility was capped at 3000 m for both sites, and we decided to use the visibility value of the VAISALA system after comparing CCTV images of both sites.

**Figure 3:**
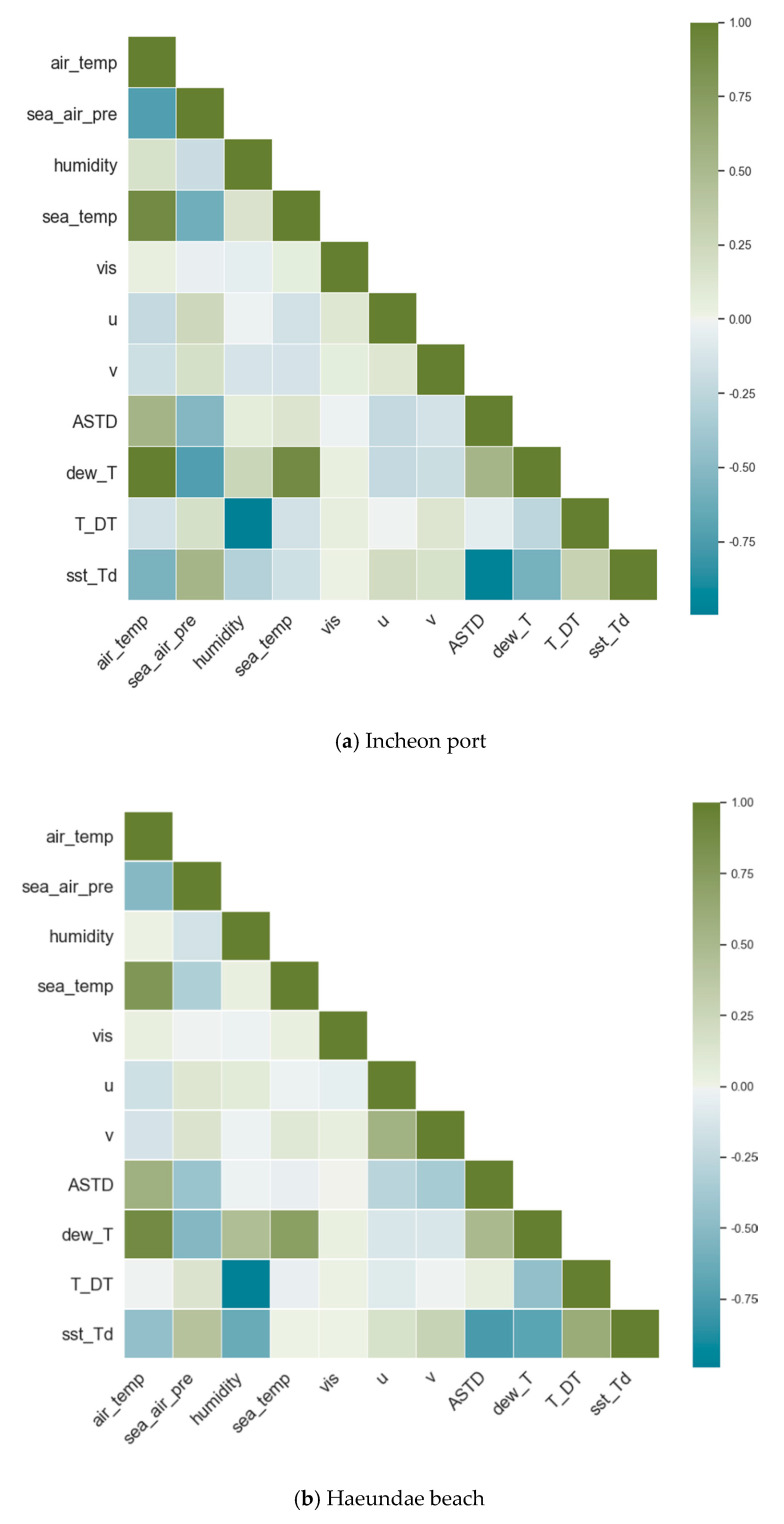
Pearson correlation matrix of the 11 features for (**a**) Incheon port and (**b**) Haeundae beach.

Weather conditions are related to each other, and despite a high variance in each individual feature, there is a considerable correlation among some features, as shown in Figure 3. Air temperature has a strong correlation with sea surface temperature, air and sea temperature difference (ASTD), and sea surface temperature and dew point temperature difference (sst_DT). Air temperature is a component of the latter two and, therefore, a high correlation is expected. There are strong and negative correlations between air temperature and sea surface pressure, and sea surface temperature and dew point temperature differences (sst_DT). Again, the latter feature is derived from air temperature, and therefore a high correlation is inevitable.

There were other correlations, both positive and negative, among the rest of the features in both Incheon port and Haeundae beach. Despite these highly correlated features, due to the low count of the number of features available, all of them were included in the training. Therefore, we could not choose a linear model as a learning model. The list of base seven features was extended with derived features, such as air and sea temperature difference and dew point temperature, for the same reason. Overall, as weather conditions are highly dependent on each other, the observed correlation is inevitable.

The count of the final selected data is summarized in Table 3. Despite the shorter observation period, there are more data available for Haeundae than Incheon since fog was more frequent in Haeundae beach during the observation period. Moreover, Haeundae beach had more missing values, and therefore more data points were removed as a result. A similar effect was observed when the past three-hour features were selected instead of the past hour-hour features: the number of missing values increased, and more data points were removed as a result.

**Figure 4:**
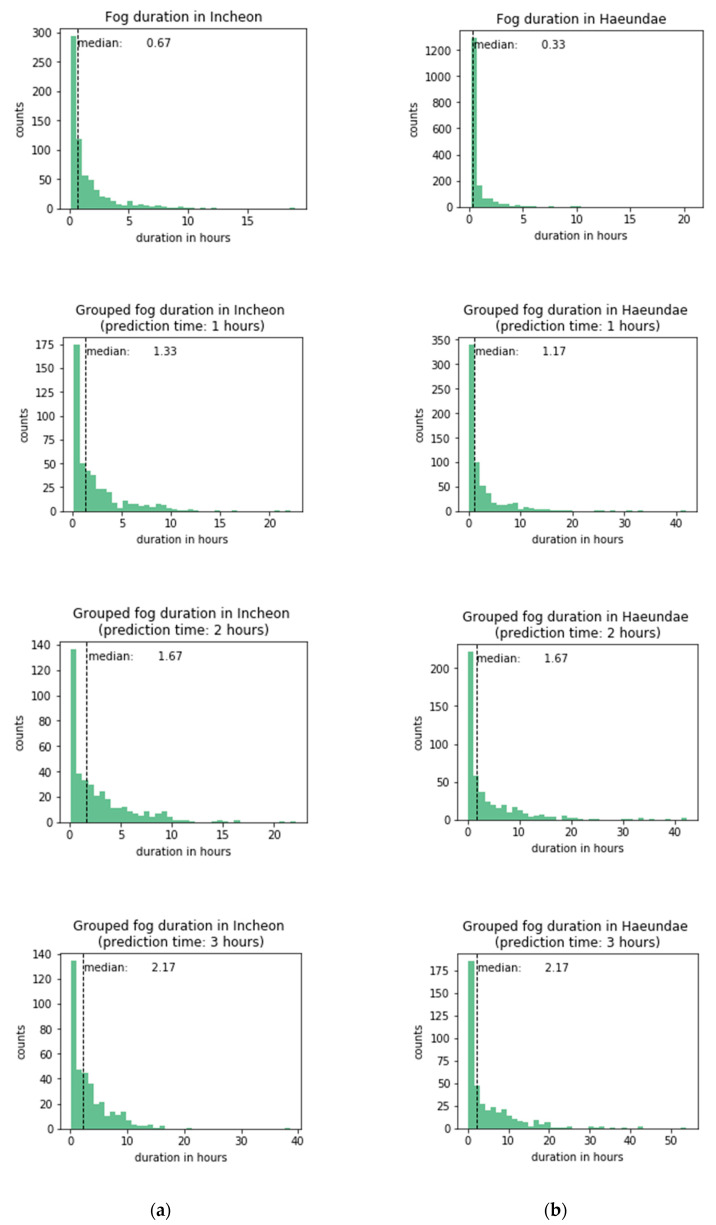
Fog duration in (**a**) Incheon port and (**b**) Haeundae beach.

As the grouping period is extended, the probability of the fog dissipation also increases and is reflected in more data being in the dissipation class than in the non-dissipation class. The grouped fog period contains observations with a visibility less than or equal to 1100 m (considering the 10% error of the optical visibility equipment) that are within one (two or three) hour apart and other observations in between them. In both of the studied sites, if not grouped, consecutive fog periods had a median of 0.67 and 0.33 h for Incheon and Haeundae, respectively (Figure 4). However, visibility fluctuates around the threshold, and fog tends to go and come back before final dissipation. Therefore, periods of fog were grouped within one-, two-, and three-hour intervals, and the median duration increased to 1.33, 1.67, and 2.17 h in Incheon port and 1.17, 1.67, and 2.17 h in Haeundae beach, respectively. On average, Haeundae has longer fog durations than those of Incheon.

**Figure 5:**
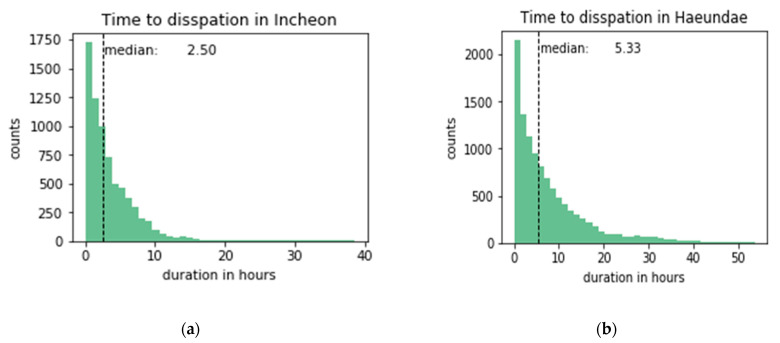
Time to dissipation in (**a**) Incheon port and (**b**) Haeundae beach.

The time to dissipation is a continual measurement that we model for predictions. Figure 5 shows the distribution of the time to dissipation for both sites. The time to dissipation was calculated by grouping fog cases that are within 3 h apart from each other. The time to dissipation was left-skewed, with half of the fog dissipation within 2.5 h in Incheon and 5.33 h in Haeundae beach. Haeundae beach hada longer time to dissipation on average than that of Incheon port and was more difficult to predict, as is discussed in Section 4.

## 3. Classification and Regression Algorithms

Each of the below-discussed methods was used for the two purposes of classification and regression. Model selection criteria were based on the simultaneous presence of a classifier and regressor (AdaBoost, bagging, extra trees, gradient boosting, random forest, k-nearest neighbors, and decision model), except for linear models and multiclass models. In addition, nine machine learning models were selected by replacing the HistGradientBoosting existing in Scikit-learn with the famous Light GBM model. Finally, since our goal was to perform predictive performance tests on deep learning models, we selected the most famous models of FFNN, CNN, and RNN. The hyperparameters used are the default ones of Scikit-learn version 0.21, unless otherwise mentioned, and they are given in the Appendix A.

### 3.1. k-Nearest Neighbors (k-NN)

k-NN is one of the non-parametric classifiers that uses the distance between a data point and its closest k neighbors to decide on the class [17]. The most represented class in the neighborhood of the data point is decided on the class of the data. The algorithm needs an appropriate number of neighbors to be selected for classification. Depending on the number of k, the algorithm may take longer or shorter, and most importantly, choose an appropriate class.

k-NN regression attempts to predict the value of the output variable by using a local average. We used default settings and deemed the number of closest neighbors to be 7.

### 3.2. Decision Tree (DT)

The decision tree classifier is one of the widely used early classification algorithms in data mining. The model is derived from the research paper on the classification and regression trees (CART) [18]. The decision tree is built by splitting the data on the basis of the Gini impurity measure, which is calculated as

(4) $$I_{g}\left(p\right)=1-\overset{J}{\underset{i=1}{\sum }}p_{i}^{2}$$

where $I_{g}$ is Gini impurity, J  is the number of classes from p∈{0,1}, and $p_{i}^{}$ is the fraction of items with label i (=sea fog dissipation). Thus, each step is decided until the minimum number of items is left with the node, which then becomes the leaf, and the splitting is then discontinued on that node.

In the case of regression, a similar tree construction algorithm is employed with a mean square error being the measure function. In both classification and regression, we did not change the default hyperparameters.

### 3.3. Support Vector Machine (SVM)

SVM represents another class of discriminative classifiers that separate data points by building a hyperplane in a dimension. Originally proposed by [19], we used the specific implementation provided in Scikit-learn [20], i.e., the C-Support vector classification algorithm. 

In practice, a support vector machine for classification and regression has been shown to perform the worst among the algorithms. We spent some time trying to tune it, but it turned out that the model itself was not well suited for the task at hand. The only hyperparameter we decided to change was the gamma option, which is the kernel coefficient for ‘radial base function’, ‘polynomial’, and ‘sigmoid’ for which we chose ‘scale’ in the Scikit-learn package. 

### 3.4. Bagging and Boosting Ensemble Models

#### 3.4.1. Random Forest (RF)

The random forest classifier is a type of randomized tree ensemble that uses an ensemble of decision trees [21]. Each decision tree is trained separately on the random subsample of the training data with replacement, and the final decision is made on the basis of the average of the trees in the ensemble.

In the experiments, we found that setting the number of estimators to 100 facilitated good performance, and we left the other hyperparameters unchanged for both classification and regression. The same hyperparameters as those in other models were applied as one value in order to make comparisons in similar states.

#### 3.4.2. Extremely Randomized Trees (ET)

An extremely randomized classifier is another type of randomized tree ensemble, very similar to the random forest classifier, except that the several decision thresholds for the splits are selected randomly, and the best threshold among these is chosen as the split threshold [22].

Similarly to the random forest, we left the default hyperparameters of extremely randomized classifiers and regressors unchanged.

#### 3.4.3. Bagging 

The bagging classifier is very similar to the random forest classifier except that it is a metaclassifier that can build its ensemble from any classifier and not only a decision tree. Given a metaclassifier, it builds several estimators that are fit to a random subsample of the data generated with replacement. The final decision is based on the average of all models’ performances [23].

For both tasks, we chose the decision tree as the base classifier. Regression is achieved in a similar fashion to that of classification.

#### 3.4.4. AdaBoost (AB)

The AdaBoost classifier is a representative classifier for model boosting, which, unlike bagging, relies on a set of weak classifiers. Each classifier is trained successively and not in parallel, concentrating on the error of the previous one. Such an ensemble of weak classifiers then produces a cascading effect with a net-positive effect on the accuracy of the final result [24]. 

#### 3.4.5. Gradient Boosting (GB)

Gradient boosting can be seen as another boosting algorithm, but it is more general than the AdaBoost classifier. The main difference between the AdaBoost classifier and gradient boosting is that the latter identifies the shortcomings of each weak classifier by its gradient [25,26]. This difference is expressed in the particular loss function of each classifier.

The learning rate was set to 0.1, and the remaining parameters were set to default. 

#### 3.4.6. Light GBM (LGBM)

While there are many boosting classifiers based on decision trees, they have the disadvantage of an extensive training time. In this regard, Light GBM classifier is 20 times faster in terms of training when compared with the other algorithms while providing almost the same accuracy [27]. This is achieved by gradient-based, one-side sampling and exclusive feature bundling.

Similar to other cases, the classification and regression models used the same hyperparameters, and no change was made to the default hyperparameters.

### 3.5. Neural Network-Based Architectures

#### 3.5.1. Feed-Forward Neural Network (FFNN)

The simplest neural network, in terms of its architecture, is a feed-forward neural network that builds a layer of perceptrons on top of another layer [28]. Such a simple architecture has been proven effective for most classification tasks. In the case in which the past time steps are required, they may be incorporated by concatenating with the current time step. For both of the tasks, we concatenated base features for each time step to the base features. Then, there were three deep layers of 512, 256, and 256 neurons that were each followed with batch normalization and ReLU activation layers. The final layer was a logistic regressor/classifier with the sigmoid activation function. In the case of the regression, the output was used as it was, while for the classification case, the results that were equal to or higher than 0.5 were treated as ones or zeros otherwise. 

#### 3.5.2. Convolutional Neural Network (CNN)

One of the earliest versions of the CNN was designed to recognize hand-written digits [29]. Although originally designed mostly for image-related tasks, the CNN has been adopted to a broad range of tasks, such as text classification, spatiotemporal data analysis, and weather forecasting. 

We modeled our classification and regression tasks as convolutions over time steps with each time step having 11 dimensional features (seven base features and four derived features). Similar to the FFNN, we concatenated each time step but did not flatten them. Thus, each input was similar to an image with just one filter. There were two convolution layers with output filter sizes of 512 and 256 that were each followed by the ReLU activation layer. The final layer was passed to a perceptron with the same activation case as in the case of the FFNN.

#### 3.5.3. Recurrent Neural Network (RNN)

Composed of LSTM cells, the RNN is able to interact with and remember long-term dependencies [30]. Therefore, unlike the FFNN and CNN, the RNN is well suited for time-series data, such as fog dissipation [31]. Unlike the other neural network architectures, inputs are not concatenated in the case of the RNN. Instead, each time step is an input with 11 features and the 1-h past features represent 6 past time steps, while 3 and 6 h represent 18 and 36 past time steps, respectively.

There are two layers of recurrent LSTM cells that are stacked. Each recurrent layer passes its output forward with the third recurrent layer returning only the last LSTM cell’s output. Each LSTM cell has 64 neurons, and the last recurrent layer’s output is passed to a perceptron just like the other two architectures with the same activation function for the classification and regression tasks.

### 3.6. Evaluation

For the binary classification task, we evaluated each model’s performance using the critical success index (CSI) score, precision score, recall score, and F1 score. The CSI score is a verification measure of categorical forecast performance equal to the total number of correct event forecasts (hits) divided by the total number of correct forecasts plus the number of misses. The precision score is the ratio of correctly predicted positive observations to the total predicted positive observation, while the recall score is the ratio of correctly predicted positive observations to the total number of events observed. The F1 score is the harmonic mean of the precision score and recall score. Given a confusion matrix, the scores are calculated as follows:

CSI score = TP/(TP+FP+FN)(5)

Precision score = TP/(TP+FP)(6)

Recall score = TP/(TP+FN)(7)

(8) $$F1\;\mathrm{score}=2\;\frac{precision\times recall}{precision+recall}$$

where TP—true positive, TN—true negative, FP—false positive, and FN—false negative.

For the binary classification task, we evaluated each model’s performance using Mean Squared Error (*MSE*), Root Mean Square Error (*RMSE*), Mean Absolute Error (*MAE*), and the coefficient of determination (*R^2^*). The mean squared error measures the average of the squares of the errors. Taking the square root of *MSE* yields the Root Mean Square Error, which has the same units as the quantity being estimated. The mean absolute error is an arithmetic average of the absolute errors. R squared represents the proportion of variance (of y) that has been explained by the independent variables in the model. All of the scores are calculated as follows:

(9) $$MSE=\frac{1}{n}\overset{n}{\underset{i}{\sum }}{\left(y_{i}-{\hat{y}}_{i}\right)}^{2}$$

(10) RMSE=√MSE

(11) $$MAE=\frac{1}{n}\overset{n}{\underset{i}{\sum }}\left|y_{i}-{\hat{y}}_{i}\right|$$

(12) $$R^{2}=1-\frac{Unexplained\;Variation}{Total\;Variation}$$

where $y_{i}$ is ground truth, ${\hat{y}}_{i}$ is the predicted value or data point at index i, and $\bar{y}$ is the mean of all values.

## 4. Results

For the experiments, we used an ordinary desktop computer with the Microsoft Windows 10 operating system, an Intel-based CPU of 2.90 GHz with two cores, NVIDIA GeForce GTX 1660 GPU accelerator, and two DDR 4 RAMs of 8 GB. We ran each model five times and report here their median performance.

### 4.1. Classification Results

When modeled as a classification task, the models showed performance that is consistent over the prediction times of one, two, and three hours, as shown in Table 4, Table 5 and Table 6, respectively. As the prediction time is extended, the prediction of dissipation (occurrences) becomes more accurate. Another dimension along which improvement is achieved is the past time steps—more features resulted in better accuracy for all models. The accuracy was higher for three-hour features as compared with the one-hour ones. The performance of the models over the prediction periods was averaged and ranked. The rankings derived in this way demonstrate relative consistency when compared to the number of past time steps. 

Among the studied sites, the predictions of Incheon port in terms of the CSI score, being the median of the models, were about 11% higher than those of Haeundae beach. The prediction accuracy improved as the prediction period was extended. The most accurate predictions came from some of the ensembles and neural network models. Extremely random trees and random forest were the strongest among the ensemble methods, while RNN was best among neural network-based classifiers. 

The SVM performed the worst among all of the models. Its performance for one-hour prediction was below the random guess in terms of the class distribution of the training data. SVM classifies on the basis of the separating planes in high dimensions, and it seems that for the fog dissipation within one hour, it could not find an accurate plane with the radial basis function (RBF) kernel. Other kernels with different gamma and penalty values did not show any improvement over the default RBF kernel. 

Another classifier that performed worse than others but better than SVM was the gradient boosting-based ensemble model. Gradient boosting builds an ensemble sequentially by using weak classifiers with each next one building on the error of the previous one. While tuning it for better performance, the number of estimators appeared to be the most important among other hyperparameters. The current results were calculated using an ensemble of 10,000 estimators.

Other non-neural network-based models demonstrated reasonable results with their default settings in Scikit-learn package [32]. Neural networks were tuned on the number of layers and the number of neurons at each layer. Among the three neural network-based architectures, the RNN was the best, except when used with the base features. When used with the base features, there are no past time steps that can recur, and therefore the model cannot learn from the past time steps. On the contrary, when the past time steps are input to the model, fog dissipation is usually best captured by the recurrent nets. Overall, among the neural network-based models, the RNN demonstrated higher performance for Haeundae beach than for Incheon port as compared with extremely random trees. 

In general, the longer the period of prediction, the more accurate the models’ performances and the less the difference between them. Using only base features is also effective in predicting fog dissipation with most of the models. Fog dissipation in Incheon port is more predictable than in Haeundae beach, although slightly more data are available for the latter. This could be due to the meteorological conditions in Haeundae beach being more complex than those in Incheon port. 

**Figure 6:**
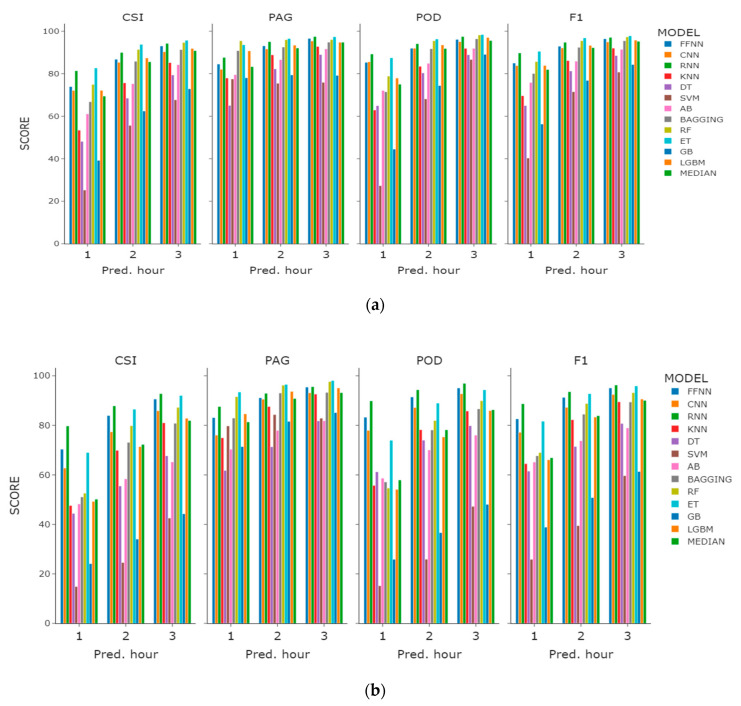
Performance comparison graph of the regression models with the highest CSI performance within the features experiment: (**a**) Incheon port; (**b**) Haeundae beach.

On the basis of the value with the highest prediction performance among base features, 1H features, and 3H features, we plotted a comparison graph for the prediction performance and the learning model selected by CSI score (Figure 6). When checking the overall performance index, we found that most of the models showed very good PAG performance, but POD performance showed large regional variations in Incheon and Haeundae. Among the tree-based models, the POD performance of the ET algorithm was the best. Learning sea fog dissipation prediction as a classification model has shown that algorithms such as FFNN, RNN, RF, and ET are superior to the other models.

### 4.2. Regression Results 

For the regression task, we grouped fog cases that are three hours apart. Then, we fit models to predict the time to dissipation given each case within the grouped fog period. Similar to the classification case, we experimented with base features, past one- and three-hour features. Unlike the classification model, the regression model showed that a larger number of features did not always result in more accurate predictions. For a number of tree-based ensemble models, such as bagging and random forest, their performance with only base features was on par with their past three hours feature models, as can be observed in Table 7.

Among the neural network-based models, the FFNN was the most accurate, with the convolutional and recurrent architectures being much less accurate. Given three hours of past features, FFNN presented an ***R^2^*** of 0.99 and 0.97 for Incheon port and Haeundae beach, respectively, and this ranks it as the best model. Given base and past one-hour features, it ranks as the second-best for Incheon port and fourth and second for Haeundae beach. Recurrent neural nets demonstrated the worst performance of 0.04 among the models given base features, which is not surprising since the prediction was made only with a single LSTM cell at each recurrent layer. Overall, except for the FFNN, both the RNN and CNN ranked much worse than the other models when compared with their ranking in the classification case.

Extremely random trees performed the best in most of the cases, just as in the classification case. Other tree-based ensemble models, such as bagging, random forest, and Light GBM, performed on par with each other. The worst performance was demonstrated by AdaBoost and SVR, with k-nearest neighbors performing slightly better. Overall, the regression results appeared to be quite accurate, and the dissipation time was predicted within reasonable accuracy ranges.

**Figure 7:**
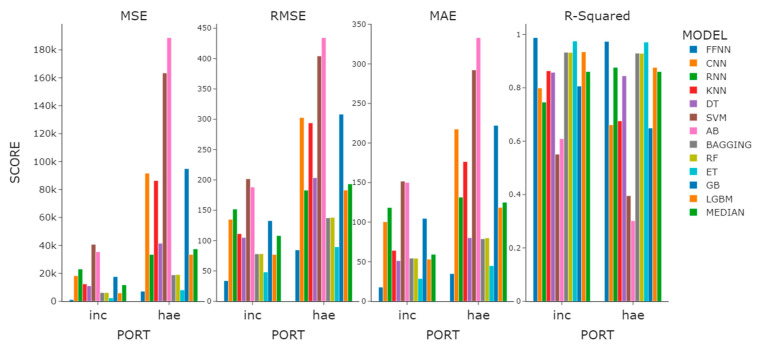
Performance comparison graph of the regression models with the highest ***R^2^*** performance within the feature experiment.

Observing the median value of the overall performance of the regression models for sea fog dissipation, we found the ***R^2^*** values of Incheon and Haeundae to appear to be similar, but the MSE, RMSE, and MAE of Incheon were observed to be lower by more than half when compared with those of Haeundae (Figure 7). What is noteworthy here is that the RNN model, which did not have a large difference in classification model prediction performance in the features experiment, distinctively differed in prediction performance in the regression models. The ***R^2^*** difference between the model using base features and the model using 3H features was 0.84. We confirmed once again that time-lagged data must be used when using the RNN model as a regression model.

## 5. Discussion

The tree-based ensemble models, together with neural network-based models, demonstrated relatively high performance on both classification and regression tasks. In the classification task, higher prediction accuracy was achieved when the period of prediction lengthened from one hour to three hours. This was partly expected as more of the cases fell under the dissipation case rather than non-dissipation as the prediction period was extended. 

The length of fog duration rose as the period of prediction increased since we grouped fog cases that were within the prediction time frame. In Incheon port, the median duration of fog was 0.67 h initially, and this doubled to 1.33 h under one-hour prediction and rose to 1.67 and 2.17 h under two- and three-hour prediction regimes, respectively. For Haeundae beach, median fog duration started at 0.33 h and then rose to 1.17, 1.67, and 2.17 h as the prediction period was extended. In both sites, as we grouped fogs that were within one, two, and three hours, the median fog duration rose but not at the same pace as the prediction time frame. This influenced non-dissipation and dissipation class distribution. For Incheon under a one-hour prediction regime, 70% of the cases fell within the non-dissipation class and 30% within the dissipation class. When the prediction period was extended to three hours and the fog cases that were grouped were within three hours, the distribution of the prediction classes changed, with 45% of the data falling within the non-dissipation class and the rest within dissipation. Under the three-hour prediction regime, the most extreme cases were grouped and fell within non-dissipation classes. Such cases were outliers, more exceptional, and occurred under more differentiated weather conditions. This should make detecting non-dissipation cases easier than under the one- or three-hour regimes, and similar assumptions should hold for the case of Haeundae beach.

For the classification task, the reason why the predictive performance of the ET model was high is thought to be because the decision boundary was generalized through extreme random variable modeling for a slight overfitting caused by a small amount of training data. The reason the SVM and AB algorithms showed low predictive performance was considered to be due to the fact that the learning model was not sufficiently optimized with the default hyperparameters.

For the regression task, the three-hour prediction regime was selected as the basis for grouping fog cases that happened within this time frame. This made the median and maximum time to dissipation 2.50 and 38.50 h for Incheon, and 5.33 and 53.66 h for Haeundae beach, respectively. As the predictions were made for grouped fog cases under this time frame, we do not know how the results would be if other time frames were chosen. Each model’s performance varied in terms of the number of features used, but the increase in the accuracy was not always achieved as more time steps were included in the input. The increase was positive for neural network-based models, k-nearest neighbors, SVR, and some tree-based ensemble models. An increase is expected when the models learn to condition on the past and perform more accurately. This was most evident in the case of recurrent and convolutional neural nets. However, in the case of the random forest and decision tree, more features confused the models with a net drop in *R^2^*. Similar observations for the same classifiers were observed in the classification case under a one-hour prediction regime. Overall, more features meant better performance for most of the cases for classification and regression tasks. 

Despite relatively few base features and high intercorrelation for some of them, the outcome of the experiments demonstrated accurate results due to the use of machine learning models. Higher performance may be achieved by more weather data included in the base features and a longer observation time span. The latter is especially beneficial in the case of neural network-based models, as they thrive on large amounts of training data. Another dimension for improvement is to include other models with different architectures, such as transformers [33], logistic or linear regression, lazy learning (e.g., one of the time series prediction models) [34], and/or other machine learning methods.

## 6. Conclusions

In this work, we addressed fog dissipation prediction in Incheon port and Haeundae beach of the Korean peninsula. Fog dissipation is a relatively under-researched area, with more research available on fog prediction and forecasting. This causes the current research results to be less comparable with previous research, and no benchmarking datasets were used to compare the results. 

The Korean peninsula is in East Asia, and the weather contrast is more distinct from north to south and from east to west due to the distinct geography of its location. Two studied sites that are important for the Korean economy and recreation, Incheon port and Haeundae beach, are located in the northwest and southeast of the country, respectively. Fog is more frequent in Haeundae beach and lasts longer than in Incheon port. Through the experiments, we found that this was also reflected in the less accurate predictions for Haeundae beach than those for Incheon port. 

Our results demonstrate high prediction accuracy when dissipation prediction is modeled as classification and regression tasks. CSI scores were within 0.82 and 0.96 for the best models of classification depending on the prediction horizon for the classification task. The score was higher when the prediction period was longer and when more past time steps were included. Regression accuracy was also improved when past time steps were included, but not for all models. The best model’s ***R^2^*** ranged from 0.93 to 0.99 for Incheon port and from 0.93 to 0.97 for Haeundae beach, depending on the past time steps used for prediction, as shown in Table 7.

## Figures and Tables

**Table 1:** Eleven features (seven base features and four derived features).

Item	Variable Name	Unite of Measurement	Note	Observation Frequency
Time	Date	timestamp	n/a	n/a
Air temperature	*air_temp*	°C	feature 1	1 min
Sea surface pressure	*sea_air_pre*	hPa	feature 2	1 min
Relative humidity	*Humidity*	%	feature 3	1 min
Sea surface temperature	*sea_temp*	°C	feature 4	1 h
Visibility	*Vis*	m	feature 5	1 min
U wind (10 m)	*U*	m/s	feature 6	1 min
V wind (10 m)	*V*	m/s	feature 7	1 min
Air and sea Temperature difference	*ASTD*	°C	feature 8	1 min
Dew point temperature	*DT*	°C	feature 9	1 min
Air and dew point temperature difference	*T_DT*	°C	feature 10	1 min
Sea surface temperature and dew point temperature difference	*sst_DT*	°C	feature 11	1 min
Dissipation	*L*	[0,1]	label	n/a
Time to dissipation	*ttd*	minutes	continuous target	10 min

**Table 2:** Data statistics for Incheon and Haeundae.

Variables	(a) Incheon (1 January 2012–31 May 2019)					(b) Haeundae (1 Janury 2014–31 July 2019)				
	Average	Median	Std	Min	Max	Average	Median	Std	Min	Max
***air_temp***	11.28	10.40	7.84	−6.60	27.50	18.38	19.10	4.60	0.50	29.30
***sea_air_pre***	1012.21	1012.30	7.16	987.70	1035.50	1009.13	1029.10	5.83	992.50	1029.10
***humidity***	96.91	98.80	4.55	46.20	99.90	92.15	100.00	9.52	20.40	100.00
***sea_temp***	10.80	8.30	6.61	1.10	25.90	17.26	28.7	3.71	11.30	28.70
***vis***	683.91	541.00	642.66	28.40	3000.00	1048.64	750.00	923.27	10.00	3000.00
***u***	−0.81	−0.79	1.57	−7.99	6.04	−0.20	−0.08	3.26	−11.61	11.98
***v***	−0.35	−0.46	1.82	−7.73	9.87	0.07	−0.04	3.36	−14.02	12.76
***ASTD***	0.49	1.00	3.37	−14.70	9.60	1.12	1.20	2.84	−12.60	8.80
***DT***	10.79	10.04	8.00	−12.79	27.33	16.97	17.66	5.14	−9.38	26.55
***T_DT***	0.50	0.18	0.83	0.01	12.15	1.41	0.78	2.20	−0.00	20.85
***sst_DT***	0.02	−9.14	3.51	−9.14	21.69	0.28	−0.17	3.54	−8.31	1.36
***ttd***	231.27	0.00	278.53	0.00	2310.00	484.40	320.00	521.32	0.00	3220.00

**Table 3:** Input data counts for (a) Incheon (period 1 January2012 to 31 May 2019) and (b) Haeundae (period 1 January 2014 to 31 July 2019). Data counts with past one- and three-hour features. n/diss and diss denote non-dissipation and dissipation, respectively.

	Predicting Fog Dissipation Within								
	1 h			2 h			3 h		
	n/diss	Diss	Total	n/diss	Diss	Total	n/diss	Diss	Total
**(a) Incheon**									
**base** **features**	4598	1975	6573	3891	3024	6915	3247	3962	7209
	(69%)	(31%)		(57%)	(43%)		(45%)	(55%)	
**1-h** **features**	4332	1850	6182	3641	2872	6513	3007	3768	6775
	(70%)	(30%)		(55%)	(45%)		(44%)	(54%)	
**3-h** **features**	3755	1630	5385	3123	2540	5663	2575	3331	5906
	(70%)	(30%)		(55%)	(45%)		(43%)	(57%)	
**(b) Haeundae**									
**base** **features**	6848	2204	9052	7320	2918	10,238	7294	3658	10,952
	(75%)	(25%)		(71%)	(29%)		(66%)	(34%)	
**1-h** **features**	6474	2080	8554	6908	2765	9673	6830	3492	10,322
	(75%)	(25%)		(71%)	(29%)		(66%)	(34%)	
**3-h** **features**	5886	1876	7762	6228	2566	8794	6119	3222	9341
	(76%)	(24%)		(71%)	(29%)		(65%)	(35%)	

**Table 4:** Performance of classification models (one-hour).

Model Name		Incheon				Haeundae			
	Features	CSI	PAG	POD	F1	CSI	PAG	POD	F1
**FFNN**	base	63.58	75.85	80.51	77.74	55.5	69.39	74.38	71.38
	1H	65.76	80.05	78.65	79.35	58.12	72.95	73.08	73.51
	3H	73.89	84.5	85.28	84.98	70.29	83.06	83.2	82.56
**CNN**	base	53.04	64.35	78.99	69.32	44.69	53.78	69.61	61.78
	1H	61.23	73.91	78.11	75.95	51.56	68.54	68.51	68.04
	3H	72.09	82.06	85.58	83.78	62.78	75.98	77.87	77.13
**RNN**	base	60.72	73.2	77.47	75.56	51.15	63.45	73.02	67.68
	1H	72.55	85.52	83.51	84.09	65.56	77.91	80.53	79.2
	3H	81.36	87.61	89.26	89.73	79.67	87.53	89.87	88.68
**k-NN**	base	43.49	71.74	52.03	60.62	33.96	65.85	41.27	50.7
	1H	43.41	73.18	51.62	60.54	38.24	69.66	46.63	55.32
	3H	53.39	77.95	62.88	69.61	47.61	74.92	55.73	64.51
**DT**	base	48.13	65.02	64.94	64.98	44.41	61.78	61.22	61.5
	1H	42.69	61.21	60	59.84	34.8	52.67	51.2	51.64
	3H	47.45	64.31	64.72	64.36	35.88	53.1	52.53	52.82
**SVM**	base	18.99	70.53	20.76	31.92	10.4	82.54	10.66	18.84
	1H	18.18	75.61	18.92	30.77	11.19	75.71	11.54	20.13
	3H	25.21	77.5	27.3	40.27	14.84	79.71	15.2	25.85
**AB**	base	45.42	66.45	59.37	62.46	30.97	58.05	39.23	47.29
	1H	50.43	70.66	63.78	67.05	36.49	58.81	48.8	53.47
	3H	61.07	79.52	72.09	75.83	48.25	70.29	58.67	65.09
**Bagging**	base	62.61	83.24	71.65	77.01	51.12	82.89	57.14	67.65
	1H	58.25	82.52	66.76	73.62	41.7	85.12	44.71	58.86
	3H	66.76	90.8	71.47	80.07	45.36	89.19	46.93	62.41
**RF**	base	69.3	87.92	76.46	81.87	51.46	86.93	55.78	67.96
	1H	69.15	90.73	75.14	81.76	46.35	88.74	48.8	63.34
	3H	74.93	95.45	78.83	85.67	52.59	91.52	54.67	68.93
**ET**	base	76.04	88.91	83.67	86.39	61.38	88.55	66.67	76.07
	1H	81.16	92.02	86.49	89.6	62.81	91.84	66.11	77.16
	3H	82.61	93.57	87.42	90.48	68.91	93.38	73.87	81.59
**GB**	base	32.05	72.29	36.58	48.51	16.67	72.55	17.91	28.57
	1H	33.49	72.91	37.84	50.18	20.09	68.67	21.63	33.46
	3H	39.17	78.04	44.48	56.29	24.11	71.33	25.87	38.85
**LGBM**	base	57.41	81.4	65.7	72.94	41.8	81.27	46.26	58.96
	1H	60.05	85.02	68.65	75.04	40.95	81.3	45.67	58.1
	3H	72.16	90.71	77.91	83.83	49.27	84.58	54.13	66.02
**Median of** **Models**	base	55.38	73.37	69.62	71.28	44.94	70.93	55.44	62.01
	1H	58.92	79.13	67.3	74.15	41.71	74.18	48.8	58.87
	3H	68.11	83.06	75.31	81.03	49.33	82.46	57.87	66.06

**Table 5:** Performance of classification models (two-hour).

Model Name		Incheon				Haeundae			
	Features	CSI	PAG	POD	F1	CSI	PAG	POD	F1
**FFNN**	base	78.40	87.88	87.60	87.89	73.68	83.12	86.30	84.85
	1H	81.05	89.48	90.61	89.53	71.49	79.80	86.62	83.38
	3H	86.74	93.08	91.93	92.90	83.90	91.07	91.42	91.25
**CNN**	base	67.99	77.64	84.13	80.94	57.18	63.95	83.05	72.75
	1H	77.66	88.19	85.74	87.43	70.66	80.87	84.09	82.81
	3H	85.30	91.62	91.93	92.07	77.34	90.51	87.13	87.22
**RNN**	base	75.15	86.17	84.79	85.81	70.51	82.78	83.39	82.71
	1H	83.60	92.10	90.61	91.07	81.32	88.77	88.97	89.70
	3H	89.98	95.03	94.09	94.73	87.82	92.88	94.35	93.51
**k-NN**	base	66.38	82.32	76.69	79.79	54.64	79.79	63.18	70.67
	1H	66.97	82.46	77.91	80.21	57.23	81.17	65.82	72.80
	3H	75.66	88.84	83.46	86.14	69.82	87.55	78.17	82.23
**DT**	base	68.45	82.23	80.33	81.27	55.51	71.28	73.97	71.39
	1H	65.61	79.17	79.30	79.24	50.34	65.68	66.18	66.97
	3H	66.78	81.47	78.74	80.08	55.37	70.41	70.57	71.28
**SVM**	base	51.36	66.99	68.60	67.86	17.79	80.87	18.49	30.21
	1H	47.59	70.33	58.96	64.49	19.43	76.87	20.98	32.54
	3H	55.63	75.46	68.11	71.49	24.58	84.31	25.93	39.47
**AB**	base	58.08	75.13	70.58	73.48	39.04	65.81	49.32	56.15
	1H	65.17	79.86	77.91	78.91	44.79	66.14	57.14	61.87
	3H	75.26	86.60	84.84	85.88	58.37	77.87	69.98	73.72
**Bagging**	base	82.34	90.95	89.75	90.32	73.08	93.01	78.08	84.44
	1H	81.59	90.00	89.39	89.86	65.64	92.94	69.08	79.25
	3H	85.82	92.52	91.73	92.37	70.92	94.97	73.68	82.99
**RF**	base	85.14	92.82	91.90	91.97	72.26	92.95	76.71	83.90
	1H	87.87	94.16	93.22	93.54	73.06	96.44	75.05	84.44
	3H	91.38	95.98	95.47	95.50	79.81	96.18	81.87	88.77
**ET**	base	88.75	94.20	93.88	94.04	78.48	93.45	83.05	87.94
	1H	91.50	95.64	95.65	95.56	84.06	96.39	86.80	91.34
	3H	93.73	96.48	96.26	96.76	86.42	96.43	88.89	92.72
**GB**	base	56.32	73.79	70.41	72.06	27.81	82.84	29.62	43.52
	1H	56.21	73.94	70.09	71.96	29.12	77.45	32.01	45.11
	3H	62.38	79.38	74.41	76.83	34.06	81.53	36.65	50.81
**LGBM**	base	77.79	88.33	87.11	87.51	58.77	89.15	63.70	74.03
	1H	80.16	89.46	88.70	88.99	60.70	89.08	66.18	75.54
	3H	87.39	93.37	93.50	93.27	71.35	93.64	75.24	83.28
**Median of** **Models**	base	72.14	84.12	84.46	83.81	58.70	82.27	74.14	73.97
	1H	78.92	88.07	88.43	88.22	63.13	81.30	69.35	77.40
	3H	84.87	91.56	91.34	91.82	71.39	90.30	77.29	83.31

**Table 6:** Performance of classification models (three-hour).

Model Name		Incheon				Haeundae			
	Features	CSI	PAG	POD	F1	CSI	PAG	POD	F1
**FFNN**	base	84.21	92.45	90.79	91.43	78.65	85.48	90.57	88.05
	1H	86.67	92.86	92.31	92.86	79.54	87.76	88.70	88.60
	3H	93.01	96.55	96.10	96.38	90.55	95.39	95.04	95.04
**CNN**	base	75.06	86.29	85.75	85.75	64.42	71.70	86.07	78.36
	1H	84.45	93.14	90.05	91.57	76.90	84.11	89.99	86.94
	3H	90.32	95.37	95.05	94.92	85.86	93.09	92.71	92.39
**RNN**	base	81.79	90.09	90.04	89.98	74.55	83.00	89.34	85.42
	1H	90.11	95.09	94.69	94.80	87.33	94.06	92.70	93.24
	3H	94.23	97.46	97.45	97.03	92.73	95.57	96.90	96.23
**k-NN**	base	75.60	85.90	86.76	86.11	63.08	81.82	73.09	77.36
	1H	76.48	88.40	85.28	86.67	65.40	84.78	74.11	79.08
	3H	85.21	92.78	91.90	92.01	80.95	92.61	85.74	89.47
**DT**	base	79.38	88.97	88.90	88.51	67.63	81.77	79.78	80.69
	1H	75.57	84.44	86.21	86.09	57.70	73.85	73.96	73.18
	3H	77.32	86.90	87.41	87.21	59.60	76.24	73.49	74.68
**SVM**	base	58.64	71.00	74.91	73.93	23.36	75.52	25.27	37.87
	1H	61.23	71.26	81.17	75.96	28.76	78.80	31.19	44.67
	3H	67.69	75.89	86.66	80.73	42.54	82.83	47.29	59.69
**AB**	base	68.72	80.15	82.60	81.46	42.96	66.72	54.78	60.10
	1H	73.97	84.81	85.54	85.04	51.22	71.61	63.52	67.74
	3H	84.20	91.67	91.90	91.42	65.18	81.80	75.97	78.92
**Bagging**	base	88.95	92.71	95.08	94.15	80.77	93.24	86.61	89.36
	1H	89.47	92.33	96.02	94.44	77.11	95.07	80.97	87.08
	3H	91.31	94.82	96.40	95.46	80.09	96.47	81.71	88.94
**RF**	base	89.89	93.77	96.47	94.68	81.22	94.00	85.66	89.64
	1H	93.04	95.67	97.48	96.39	85.16	96.42	87.41	91.99
	3H	94.68	96.03	98.20	97.27	87.21	97.64	89.92	93.17
**ET**	base	92.64	95.42	97.10	96.18	83.77	94.44	88.11	91.17
	1H	95.12	96.86	98.14	97.50	90.79	97.31	92.13	95.18
	3H	95.64	97.32	98.35	97.77	91.98	98.03	94.26	95.82
**GB**	base	65.57	75.42	85.88	79.20	33.62	76.18	37.70	50.32
	1H	68.45	75.45	88.06	81.27	37.79	78.78	42.49	54.85
	3H	72.87	79.17	89.06	84.31	44.23	85.07	48.06	61.34
**LGBM**	base	84.13	89.81	93.44	91.38	68.06	89.37	73.63	80.99
	1H	88.67	91.57	95.89	94.00	72.36	92.47	76.39	83.96
	3H	91.85	94.73	97.00	95.75	82.79	95.06	85.89	90.58
**Median of** **Models**	base	80.66	89.18	89.03	89.30	67.84	83.21	81.28	80.84
	1H	85.72	92.03	91.71	92.31	74.36	86.15	78.83	85.29
	3H	90.70	94.64	95.65	95.12	82.03	94.33	85.81	90.13

**Table 7:** Performance of regression models.

Model Name		Incheon				Haeundae			
	Features	*MSE*	*RMSE*	*MAE*	*R^2^*	*MSE*	*RMSE*	*MAE*	*R^2^*
**FFNN**	base	5402	74	47	0.93	19,149	138	91	0.93
	1H	3071	55	36	0.96	17,787	133	80	0.93
	3H	1135	34	18	0.99	7130	84	35	0.97
**CNN**	base	37,894	195	148	0.51	184,169	429	314	0.31
	1H	29,236	171	133	0.61	166,720	408	290	0.36
	3H	18,213	135	100	0.80	91,632	303	218	0.66
**RNN**	base	62,590	250	168	0.19	255,395	505	375	0.04
	1H	38,000	195	137	0.50	128,844	359	259	0.51
	3H	23,045	152	118	0.75	33,467	183	131	0.88
**k-NN**	base	14,719	121	78	0.81	86,397	294	176	0.68
	1H	12,426	111	74	0.84	118,534	344	210	0.55
	3H	12,382	111	64	0.86	94,441	307	173	0.65
**DT**	base	11,040	105	51	0.86	41,457	204	80	0.84
	1H	19,704	140	61	0.74	58,394	242	90	0.78
	3H	16,206	127	56	0.82	74,997	274	99	0.72
**SVM**	base	54,080	233	171	0.30	191,451	438	309	0.28
	1H	45,071	212	157	0.41	179,937	424	306	0.31
	3H	40,680	202	152	0.55	163,382	404	292	0.40
**AB**	base	34,946	187	152	0.55	212,144	461	360	0.20
	1H	31,870	179	145	0.58	202,946	450	352	0.22
	3H	35,420	188	150	0.61	188,680	434	333	0.30
**Bagging**	base	5653	75	48	0.93	18,885	137	79	0.93
	1H	7048	84	57	0.91	25,154	159	91	0.90
	3H	6103	78	54	0.93	25,020	158	93	0.91
**RF**	base	5738	76	48	0.93	19,064	138	80	0.93
	1H	7126	84	58	0.91	24,644	157	91	0.91
	3H	6137	78	54	0.93	25,054	158	92	0.91
**ET**	base	3478	59	34	0.96	14,605	121	68	0.95
	1H	2681	52	31	0.96	9427	97	53	0.96
	3H	2311	48	29	0.97	8016	90	45	0.97
**GB**	base	24,558	157	122	0.68	113,195	336	245	0.58
	1H	18,605	136	106	0.75	114,988	339	248	0.56
	3H	17,593	133	105	0.81	94,924	308	222	0.65
**LGBM**	base	9135	96	72	0.88	37,383	193	141	0.86
	1H	7722	88	64	0.90	34,972	187	131	0.87
	3H	5942	77	53	0.93	33,502	183	118	0.88
**Median of** **Models**	base	13,118	114	75	0.83	64,784	251	159	0.76
	1H	15,176	123	69	0.80	89,510	296	171	0.66
	3H	12,382	111	63	0.86	64,554	254	118	0.76

## Data Availability

The data used for this study are available on request from the KHOA corresponding author. The rest of the weather data can be found at https://data.kma.go.kr/, accessed on 1 August 2021.

## Appendix A. Model Hyperparameters

CLS indicates classification model and REG indicates regression model.

**Table A1:** Hyperparameters of Sea Fog Dissipation Prediction Models.

Model Name	Incheon				Haeundae			
Parameter	CLS 1H	CLS2H	CLS 3H	REG	CLS 1H	CLS 2H	CLS 3H	REG
**FFNN**								
num_layers	3				3			
units	[521,256]				[521,256]			
**CNN**								
num_layers	2				2			
kernel_size	2				2			
units	[521,256]				[521,256]			
**RNN**								
num_layers	2				2			
units	64				64			
**KNN**								
n_neighbors	7	6	5	6	8	8	8	6
weights	distance	uniform	uniform	uniform	distance	distance	distance	uniform
**SVM**								
C	45	65	68	71	68	71	65	71
kernel	rbf	rbf	rbf	linear	rbf	linear	rbf	linear
**RF**								
max_depth	65	13	86	29	19	59	29	54
max_features	0.59	0.75	0.57	0.56	0.85	0.86	0.56	0.80
min_samples_split	4	4	5	2	4	4	2	2
n_estimators	178	219	139	365	451	460	365	423
**ET**								
max_depth	40	65	32	82	56	79	96	43
max_features	0.75	0.59	0.91	0.83	0.62	1.00	0.85	0.66
min_samples_split	3	13	4	8	3	3	9	13
n_estimators	358	359	427	339	163	128	223	219
**AdaBoost**								
algorithm	SAMME.R	SAMME.R	SAMME.R		SAMME.R	SAMME.R	SAMME.R	
learning_rate	0.94	0.69	0.85	0.59	0.99	0.84	0.80	0.45
n_estimators	412	470	370	59	499	150	102	75
**GB**								
max_depth	12	24	42	9	10	11	9	32
max_features	0.53	0.73	0.71	0.53	0.52	0.72	0.53	0.89
min_samples_split	26	9	6	14	26	6	14	16
n_estimators	386	250	498	414	400	324	414	388
subsample	0.60	0.93	0.81	0.74	0.76	0.82	0.74	0.79
**Bagging**								
bootstrap	False	False	False	False	False	False	False	False
bootstrap_features	True	False	True	True	False	True	False	True
max_features	0.69	0.52	0.63	0.52	0.81	0.86	0.62	0.53
max_samples	0.80	0.85	0.85	0.80	0.62	0.97	0.83	0.98
**DT**								
criterion	entropy	entropy	gini	friedman_mse	gini	gini	gini	mse
max_depth	246	67	475	434	351	494	354	117
max_features	0.92	0.75	0.76	0.51	0.86	0.92	0.82	0.95
min_samples_split	5	2	5	5	5	5	7	2
splitter	random	best	random	random	random	best	random	best
**LGMB**								
bagging_fraction	0.96	0.85	0.83	0.73	0.96	0.95	0.80	0.95
feature_fraction	0.62	0.68	0.85	0.66	0.96	0.86	0.63	0.72
learning_rate	0.20	0.20	0.20	0.05	0.20	0.20	0.05	0.10
num_leaves	24	30	24	20	21	23	30	26
